# Experimental study on the factors affecting torque of beam-type implant torque wrenches

**DOI:** 10.1186/s12903-021-01703-z

**Published:** 2021-07-15

**Authors:** Hiroki Shiba, Yuji Sato, Junichi Furuya, Tokiko Osawa, Akio Isobe, Myu Hayashi, Noboru Kitagawa

**Affiliations:** Department of Geriatric Dentistry, Showa University School of Dentistry, Ota Ward, Tokyo, Japan

**Keywords:** Implant, Mechanical complication, Torque wrench, Prosthetic screw

## Abstract

**Background:**

Screw breakage and loosening are the most common mechanical complications associated with implant treatment, and they may occur due to excess or inadequate screw tightening torque. When fastening and fixing the implant superstructure, screws are tightened using a torque wrench, which is essential for an accurate tightening force. However, the characteristics of the torque wrench have not been fully verified. Therefore, we aimed to clarify the factors affecting the torque with a focus on beam-type torque wrenches, which are the main types of wrenches.

**Methods:**

The torque values generated by beam-type torque wrenches from eight manufacturers were measured using a torque gauge. To investigate the influence of the location of the beam relative to the scale, measurements were performed with a scale aligned with the trailing edge, center, and leading edge of the beam respectively. Additionally, measurements were taken at 90°, 60°, and 30° to examine the effect of the angle at which the examiner read the torque value. Under each condition, a single examiner applied the recommended torque to each manufacturer's screws five times in a clockwise direction. The average measured torque, standard deviation, bias, and coefficient of variation were calculated and compared accordingly.

**Results:**

Wrenches from six manufacturers demonstrated excellent accuracy for measurements at the center of the beam (bias within ± 4%). For measurements at 90°, equipments from five manufacturers displayed excellent accuracy (bias within ± 7%), and seven showed excellent repeatability (coefficient of variation ≤ 2%).

**Conclusion:**

The scale should be aligned with the center of the beam and read from 90° while using a torque wrench. The accuracy and repeatability torques generated by the wrenches differed according to the manufacturer, scale width, scale line width, beam width, and distance between the scale and beam center. Based on these results, we suggest that a torque wrench must be selected after determining the difference in the structure of the torque wrench.

## Background

Oral implants are widely used in prosthodontic treatment [1–4]. Although implant treatment has a high success rate of > 90%, mechanical complications can occur [5], with one of the most common complications being screw breakage and loosening [6, 7]. This can lead to problems such as damage to the surrounding bone tissue and loss of osseointegration [8–12]. Factors that can cause these types of mechanical complications include incompatibility between the abutment and superstructure, repeated bending movements, initial loosening of the tightened screw, insufficient screw strength, and insufficient or excessive tightening force applied to the screw [13]. In particular, forces applied to screws are either frequently inadequate or excessively tightening and are affected by a variety of factors. This study focused on insufficient or excessive screw tightening force, which is a typical mechanical complication.

During the final fixation of the implant superstructure, the screws were tightened using a torque wrench. The use of a torque wrench is essential for an accurate tightening force [14].

Owing to the different implant and abutment connection designs, the recommended insertion torques are different [15–17]. Fixing screws are especially difficult, and large fluctuations in the applied torque have been measured in vitro. To this end, torque wrenches of various designs have been introduced accordingly[18]. Applying controlled torque allows for long-term functional loading of components. Torque wrenches should accurately display the torque applied to avoid complications during surgical and prosthetic procedures [19–21].

Torque wrenches are broadly classified into two types: mechanical and digital, with the former being further classified into beam and preset types. Various designs for manual torque wrenches are available. A digital torque wrench displays the torque digitally, a preset-type wrench stops applying force when a preset torque is reached, and a beam-type wrench passively displays the torque reached accordingly.

By comparing different designs from the same manufacturer, high torque values were obtained for the preset and beam-type devices [22]. Beam-type devices showed a more consistent range of values [22, 23]. A manual torque wrench has a mechanical design that requires proper maintenance [24, 25]. A study involving new unused devices and older devices used under normal clinical conditions showed significant fluctuations above and below the set torque value. [16, 25]

In addition, the torque exerted by an industrial torque wrench is affected by the type and structure of the torque wrench and the position of the examiner [26].

According to recent data, the variations depend on the device design and the torque level selected.

However, properties such as the accuracy and repeatability of mechanical handheld torque wrenches for oral implants have not been fully verified.

This study aimed to contribute to the long-term prognosis of implant treatment by clarifying the factors affecting torque exertion. The null hypothesis was that the torque wrench was unaffected by the operator's reading angle and beam location.

## Methods

### Materials

**Fig. 1:**
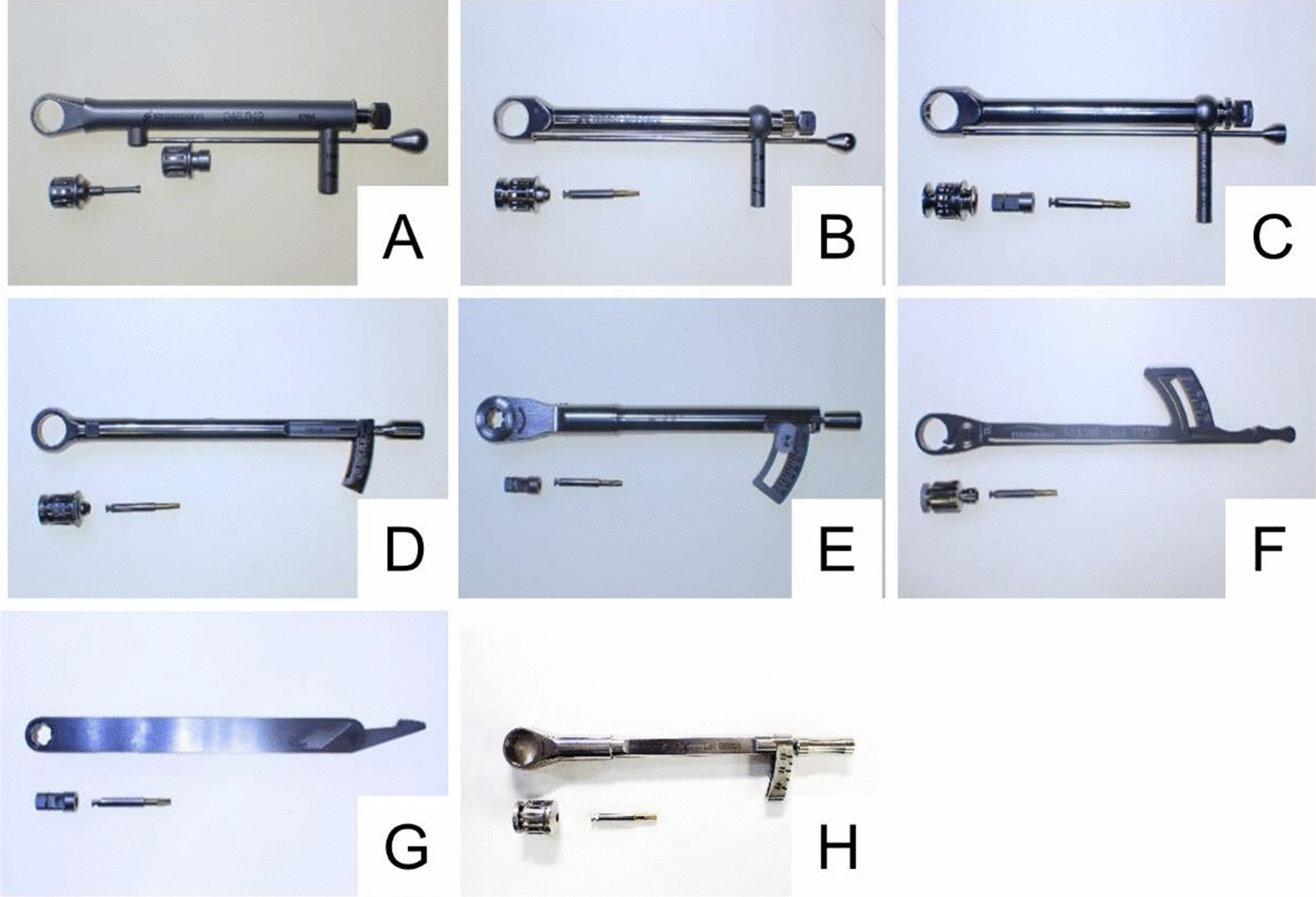
Torque wrenches studied. **a** Ratchet, Institute Straumann Ag, Basel, Switzerland. **b** Manual Torque Wrench Prosthetic, Nobel Biocare, Zürich-Frughafen Switzerland. **c** Ex Torque Wrench, Kyocera Medical Corporation, Osaka, Japan. **d** GC Implant Re and Surgical Instrument Torque Wrench, Gc, Tokyo, Japan, **e** Torque Ratchet Wrench, Ktc, Kyoto, Japan. **f** Mono torque ratchet, Thommen, Grenchen, Switzerland. **g** Torque wrench, Nippon Piston Ring Co, Saitama, Japan. **h** Biofix Torque wrench, Shofu, Kyoto, Japan

Considering the global market share, the following eight beam-type wrenches were selected: Ratchet (Institut Straumann Ag, Basel, Switzerland), Manual Torque Wrench Prosthetic (Nobel Biocare, Zürich-Frughafen, Switzerland), ex torque wrench (Kyocera Medical Corporation, Osaka, Japan); GC Implant Re and Surgical Instrument Torque Wrench (Gc, Tokyo, Japan), Torque Ratchet Wrench (Ktc, Kyoto, Japan); mono torque ratchet (Thommen, Grenchen, Switzerland), torque wrench (Nippon Piston Ring Co., Saitama, Japan), and Biofix Torque wrench (Shofu, Kyoto, Japan) (Fig. 1). All torque wrenches were new and previously unused.

#### Choosing a torque wrench

Although many types of torque wrenches exist, beam-type torque wrenches exhibit the smallest deviations from the set values [22, 23]. We considered it clinically relevant to clarify the characteristics of beam-type wrenches used in clinical practice. Thus, in this study, we selected eight torque wrenches that are often used clinically in implant treatment, taking into consideration their market share.

### Measurement device

**Fig. 2:**
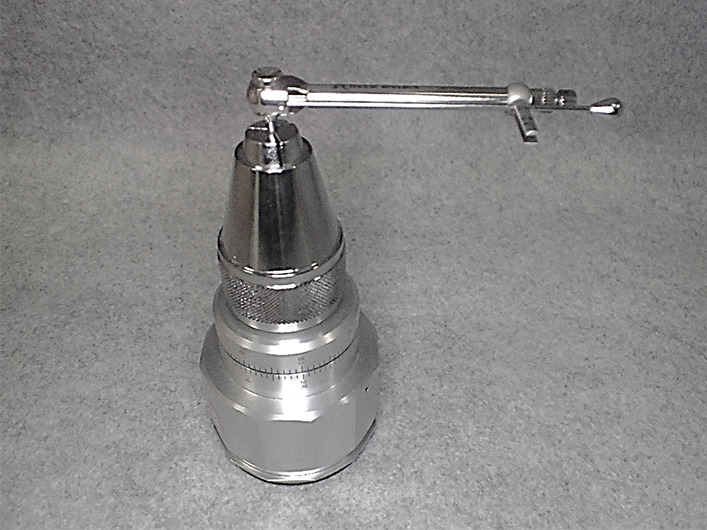
Torque wrench and torque gauge fixed

A screwdriver (Screwdriver Machine Unigrip 20 mm, Nobel Biocare, Japan) and a torque gauge (BTG36CN, Tohnichi, Japan) were fixed (Fig. 2), and the torque exerted by each torque wrench (actual measured torque values) was measured using a Latin square design.

### Measurement of the torque value

The same examiner, who was experienced in implant treatment, applied the recommended torque to each manufacturer’s prosthetic screws (target torque value) five times in a clockwise direction (Table 1). The average of the five measured torque values (average measured torque value) was calculated and recorded accordingly. The bias, which was the difference between the average measured and target torque values divided by the target torque value, was used as an index of accuracy. The coefficient of variation, which was the standard deviation of the measured torque value divided by the average measured torque value, was used as an index of repeatability.

**Table 1:** Target torque value (recommended torque value for prosthetic screws of each company)

Manufacturer	Recommended torque value for prosthetic screw (N cm)
Straumann	15
Nobel Biocare	15
Kyoucera	20
GC	10
KTC	15
Nippon Piston Ring	20
THOMMEN	15
SHOFU	10

#### Measurement of the part of the beam to be aligned

**Fig. 3:**
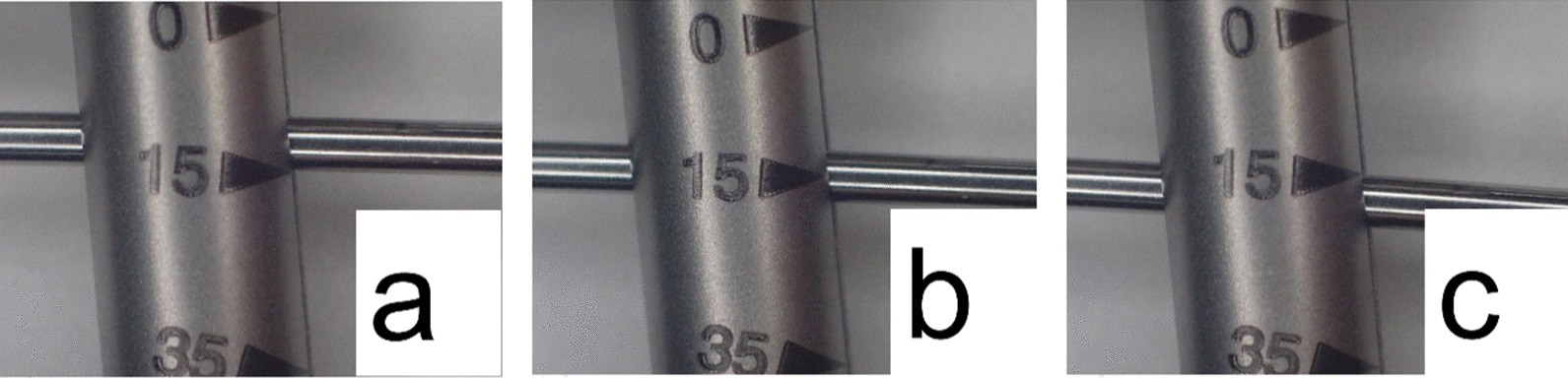
Location of the beam on the scale. **a** leading edge, **b** center, **c** trailing edge

To investigate the influence of the location of the beam relative to the scale, measurements were performed with a scale aligned with the trailing edge, center, and leading edge of the beam (Fig. 3). We analyzed whether the structure of the torque wrench influenced the part of the beam to be aligned to the scale. Thus, the torque value per millimeter of the scale, width of the scale line, and width of the beam were measured and compared with the bias and the coefficient of variation. For statistical analysis, a paired t-test was performed with Bonferroni correction. Depending on the part of the beam studied, the calculated bias for the leading edge, center, or trailing edge of the beam was used as the dependent variable for accuracy. Similarly, the coefficient of variation of the leading edge, center, or trailing edge of the beam was used as the dependent variable for repeatability according to the position of the scale on the beam. The significance level was set at 5%. Additionally, the difference in torque values between the leading and trailing edges, and the coefficient of variation in the center, were set as dependent variables. Pearson’s correlation coefficient was calculated between each dependent variable and the following three items: torque value per millimeter of scale, beam width, and scale line width. The significance level was set at 5% (Tables 2, 3). IBM SPSS Statistics 25.0 (IBM, Chicago, USA) was used for the statistical analyses.

**Table 2:** Correlation coefficients for items related to accuracy depending on the part of the beam aligned with the scale

Manufacturer	Difference in lower and upper (N cm)	Torque difference between leading and trailing edges(N cm/mm)	Beam width (mm)	Scale line width (mm)
KTC	2.1	3.3	0.1	0.2
THOMMEN	2.3	2.8	0.2	0.1
Nobel Biocare	3.4	3.6	1.0	0.1
Kyocera	4.0	3.6	1.5	0.2
Straumann	4.9	4.4	1.1	0.1
Nippon Piston Ring	5.5	4.5	–	–
GC	7.6	5.0	0.8	0.5
SHOFU	8.8	5.0	2.0	0.5

**Table 3:** Correlation coefficients for items related to repeatability depending on the part of the beam aligned with the scale

Manufacturer	Coefficient of variation (center) (%)	Torque per 1 mm in scale (N cm/mm)	Beam width (mm)	Scale line width (mm)
Nippon Piston Ring	0.8	4.5	–	–
Kyocera	1.6	3.6	1.5	0.2
Nobel Biocare	1.7	3.6	1.0	0.1
THOMMEN	1.9	2.8	0.2	0.1
KTC	2.5	3.3	0.1	0.2
Straumann	2.6	4.4	1.1	0.1
SHOFU	2.9	5.0	2.0	0.5
GC	4.3	5.0	0.8	0.5

#### Effect of the angle at which the examiner read the torque value

**Fig. 4:**
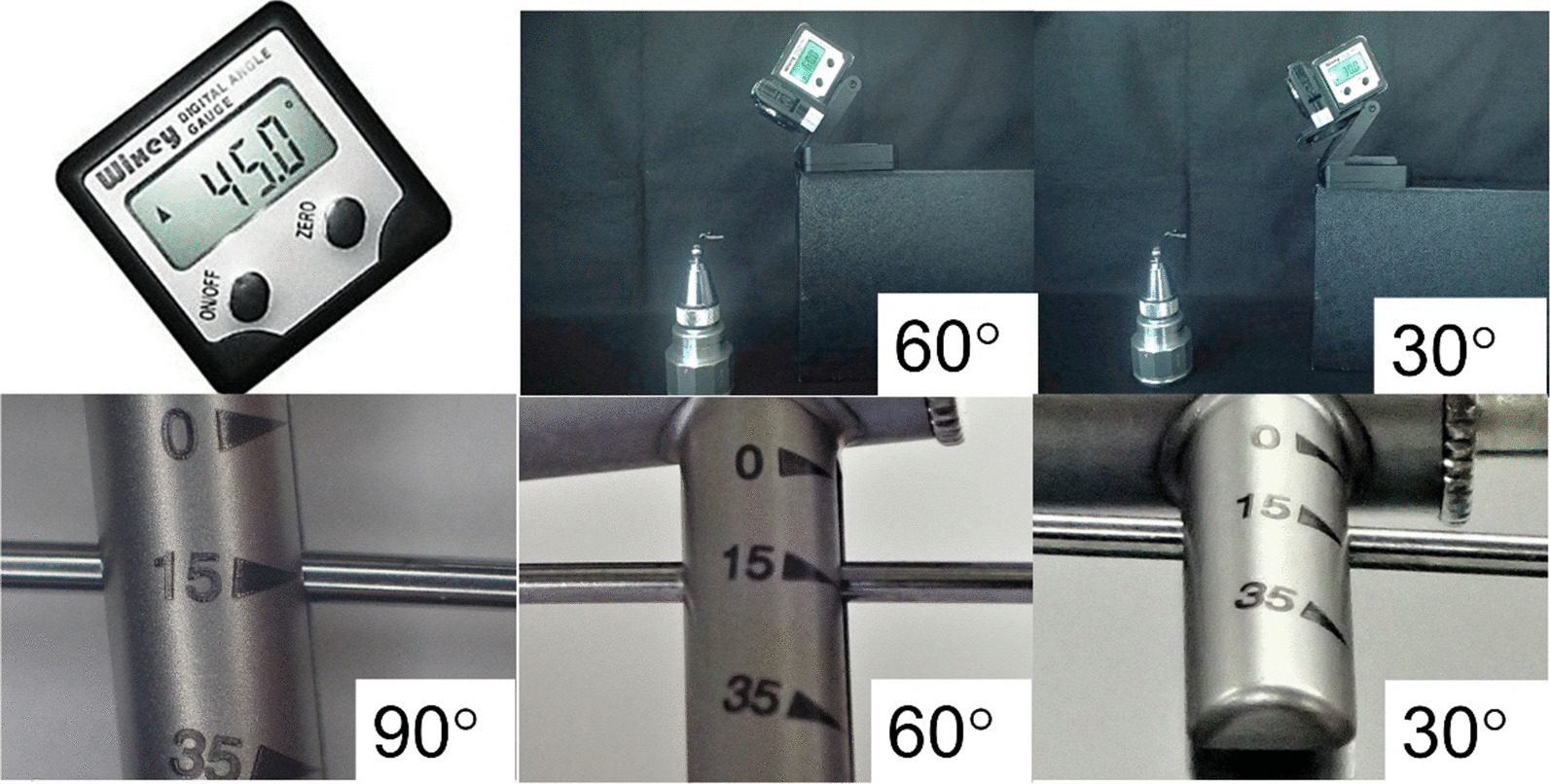
Angle at which the examiner reads the torque value

Measurements were recorded at 90°, 60°, and 30° to examine the effect of the angle at which the examiner read the torque value (Fig. 4). We analyzed whether the structure of the torque wrench influenced the angle at which the examiner read the torque value. Thus, the width of the scale line, the width of the beam, and the distance between the scale and center of the beam were measured and compared with the bias and the coefficient of variation. For statistical analysis, a paired t-test was performed with Bonferroni correction. In terms of both accuracy and repeatability, depending on the angle at which the examiner read the torque value, deviations of 90°, 60°, and 30° were used as dependent variables. The significance level was set at 5%. Additionally, the difference in torque values between 90° and 60° and the coefficient of variation when viewed from 60° were set as dependent variables. Pearson’s correlation coefficient was calculated between each dependent variable and the following three items: beam width, scale line width, and distance between scale and beam center. The significance level was set at 5% (Tables 4, 5). IBM SPSS Statistics 25.0 (IBM, Chicago, USA) was used for the statistical analyses.

**Table 4:** Correlation coefficient for items related to accuracy depending on the angle at which the examiner read the torque value

Manufacturer	Difference in torque between 90° and 60 (N cm)	Torque per 1 mm in scale (N cm/mm)	Distance between scale and center of beam (mm)	Scale line width (mm)
KTC	0.3	3.3	$\fallingdotseq$0	0.2
THOMMEN	0.9	2.8	$\fallingdotseq$0	0.1
Nippon Piston Ring	1.2	4.5	$\fallingdotseq$0	–
GC	1.4	5.0	0.5	0.5
Straumann	1.5	4.4	2.0	0.1
Nobel Biocare	2.1	3.6	2.0	0.1
Kyocera	2.5	3.6	2.0	0.2
SHOFU	3.3	5.0	3.0	0.5

**Table 5:** Correlation coefficients for items related to repeatability depending on the angle at which the examiner read the torque value

Manufacturer	Coefficient of variation (60°) (%)	Torque per 1 mm in scale (N cm/mm)	Distance between scale and center of beam (mm)	Scale line width (mm)
Nippon Piston Ring	1.7	4.5	$\fallingdotseq$0	–
THOMMEN	1.8	2.8	$\fallingdotseq$0	0.1
Kyocera	1.8	3.6	2.0	0.2
KTC	2.1	3.3	$\fallingdotseq$0	0.2
Nobel Biocare	2.5	3.6	2.0	0.1
Straumann	3.5	4.4	2.0	0.1
GC	4.5	5.0	0.5	0.5
SHOFU	6.0	5.0	3.0	0.5

## Results

### Comparison of the accuracy and repeatability depending on the part of the beam aligned with the scale

**Fig. 5:**
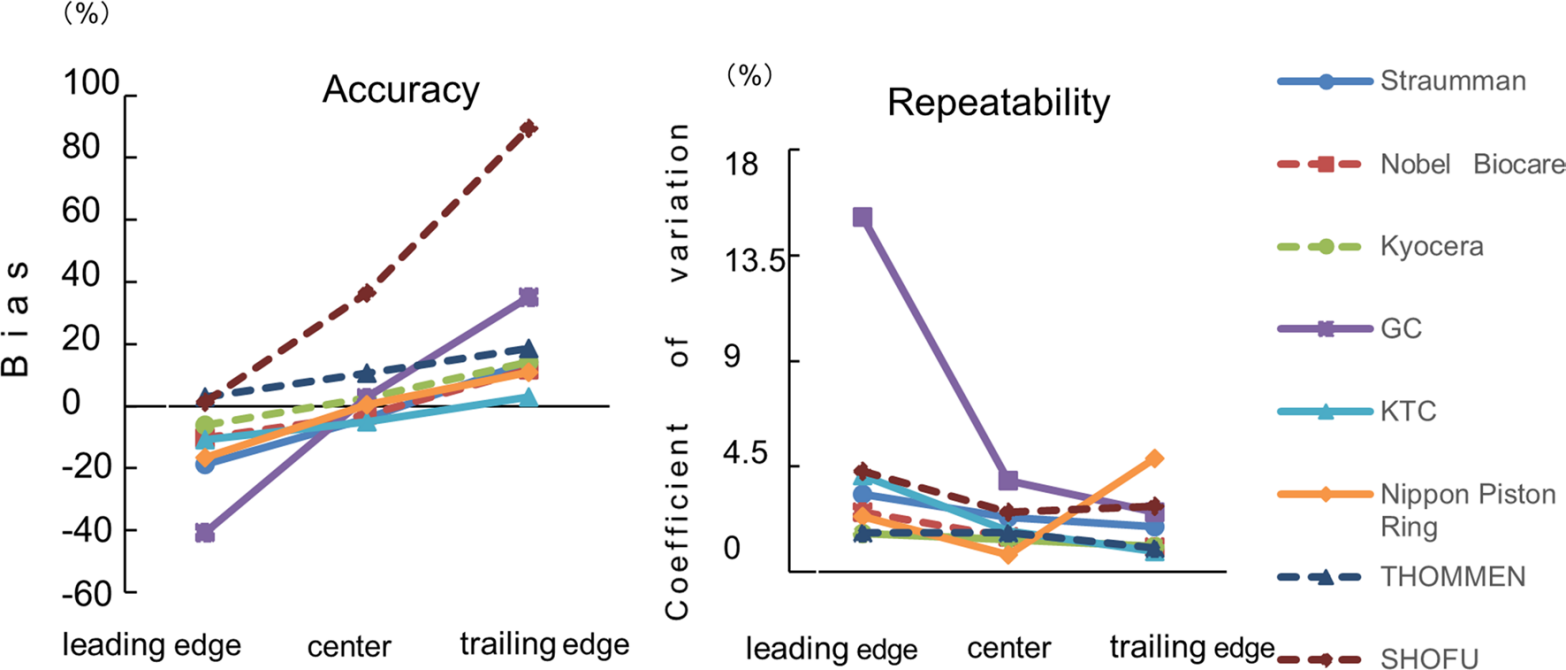
Comparison of the accuracy and repeatability depending on the part of the beam aligned with the scale

The bias and the coefficient of variation, which are indicators of the accuracy of various torque wrenches, were used for comparison (Fig. 5, Table 6). There was a significant difference in bias between the groups; however, the coefficient of variation was not significantly different between the groups (*P* > 0.05). For the wrenches from five manufacturers, the highest accuracy and repeatability (bias within ± 4%) was observed when the center of the beam was aligned with the center of the scale. As the alignment of the scale shifted from the leading edge to the trailing edge of the beam, the exerted torque tended to increase (difference in the maximum average measured torque value: ± 9 N cm). There was a significant difference in bias between the leading and trailing edges of the same manufacturer, from 12% for the smallest difference to 88% for the largest difference. Wrenches with a greater difference in bias between the leading and trailing edges demonstrated higher values of torque per millimeter scale, width of the beam, and width of the scale line. The correlation coefficient for the difference in torque values between the leading and trailing edges and the torque value per millimeter of the scale, beam width, and scale line width were 0.94, 0.57, and 0.72, respectively (Table 2). Additionally, we observed a tendency for the values of torque per millimeter of the scale, beam width, and scale line width to increase for wrenches with a higher coefficient of variation in the center. The correlation coefficients between the central coefficient of variation and the torque value per millimeter of the scale, beam width, and scale line width were 0.47, 0.29, and 0.83, respectively (Table 3).

**Table 6:** Comparison of the accuracy and repeatability depending on the part of the beam aligned with the scale

Manufacturers	Target torque value (N cm)	Part of the beam aligned	Bias (%)	Minimum	Maximum	Mean	SD	Coefficient of variation (%)
Strauman	15	Leading edge	− 18.67	11.5	12.7	12.20	0.45	3.66
		Center	− 4.00	14.0	14.9	14.40	0.37	2.60
		Trailing edge	14.27	16.7	17.8	17.14	0.37	2.14
Nobel Biocare	15	Leading edge	− 10.27	13.0	13.9	13.46	0.38	2.85
		Center	− 2.80	14.3	15.0	14.58	0.24	1.65
		Trailing edge	11.87	16.5	17.0	16.78	0.19	1.16
Kyocera	20	Leading edge	− 6.00	18.2	19.2	18.80	0.34	1.81
		Center	2.60	19.9	20.8	20.52	0.32	1.55
		Trailing edge	14.20	22.3	23.1	22.84	0.28	1.23
GC	10	Leading edge	− 40.60	4.0	6.8	5.94	1.00	16.80
		Center	2.80	9.7	10.8	10.28	0.45	4.33
		Trailing edge	35.00	12.8	13.9	13.50	0.39	2.85
KTC	15	Leading edge	− 10.67	12.2	13.8	13.40	0.61	4.55
		Center	− 5.07	13.9	14.6	14.24	0.27	1.92
		Trailing edge	3.07	15.2	15.6	15.46	0.15	0.97
Nippon Piston Ring	20	Leading edge	− 16.60	16.1	17.1	16.68	0.44	2.61
		Center	0.50	19.8	20.3	20.10	0.17	0.83
		Trailing edge	10.90	20.9	24.0	22.18	1.19	5.37
THOMMEN	15	Leading edge	3.07	15.0	15.8	15.46	0.29	1.86
		Center	10.53	16.0	16.8	16.58	0.31	1.88
		Trailing edge	18.53	17.5	18.0	17.78	0.20	1.15
SHOFU	10	Leading edge	1.40	9.5	11.0	10.14	0.48	4.77
		Center	35.80	12.9	14.0	13.58	0.39	2.85
		Trailing edge	89.40	18.2	19.9	18.94	0.59	3.11

### Comparison of the accuracy and repeatability according to the angle from which the examiner read the torque value

**Fig. 6:**
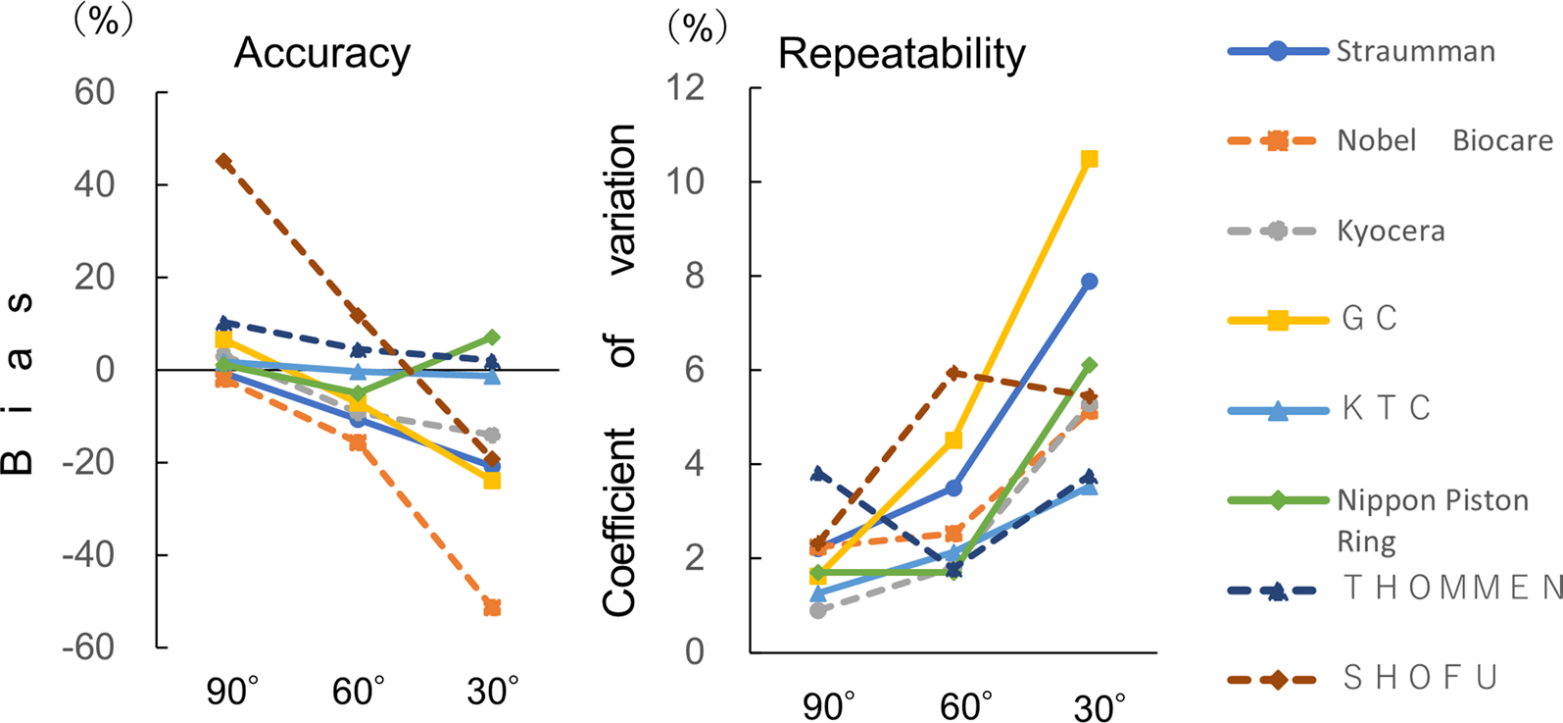
Comparison of the accuracy and repeatability according to the angle at which the examiner read the torque value

The bias and coefficient of variation, which are indices of the accuracy of various torque wrenches, were used to compare the demonstrated torque values of wrenches from seven manufacturers (Table 7). The bias was significantly different only between the 90° and 60° groups, and the coefficient of variation was significantly different between the 90° and 30° groups and the 60° and 30° groups respectively (*P* < 0.05). When the angle of torque reading was 90°, wrenches from five manufacturers demonstrated excellent accuracy (within ± 7% bias), and those from seven manufacturers showed excellent repeatability (within 2% coefficient of variation) (Fig. 6). The difference in the bias between 90° and 60° varied greatly among the manufacturers, ranging from 2% for the smallest difference to 33% for the largest difference. The correlation coefficients for the difference in torque values between the 90° and 60° angles of view and the torque value per millimeter, distance between the scale and the center of the beam, and width of the scale line were 0.45, 0.90, and 0.41, respectively (Table 4). The correlation coefficient for the coefficient of variation when viewed from 60° and the torque value per millimeter of the scale, distance between the scale and the center of the beam, and width of the scale line were 0.76, 0.57, and 0.82, respectively (Table 5).

**Table 7:** Comparison of the accuracy and repeatability according to the angle at which the examiner read the torque value

Manufacturer	Target torque value (N cm)	Angle at which the examiner read the torque value	Bias (%)	Minimum	Maximum	Mean	SD	Coefficient of variation (%)
Strauman	15	90°	− 0.53	14.5	15.4	14.92	0.33	2.22
		60°	− 10.67	12.6	13.9	13.40	0.47	3.50
		30°	− 20.80	10.9	13.0	11.88	0.94	7.89
Nobel Biocare	15	90°	− 1.87	14.2	15.1	14.72	0.33	2.25
		60°	− 15.60	12.2	13.1	12.66	0.32	2.53
		30°	− 51.20	6.6	7.6	7.32	0.38	5.14
Kyocera	20	90°	3.30	20.4	20.9	20.66	0.19	0.90
		60°	− 9.10	17.7	18.6	18.18	0.33	1.82
		30°	− 14.00	16.5	19.0	17.20	0.91	5.29
GC	10	90°	6.60	10.4	10.8	10.66	0.17	1.63
		60°	− 7.00	8.8	9.8	9.30	0.42	4.52
		30°	− 23.80	6.5	8.8	7.62	0.80	10.50
KTC	15	90°	1.87	15.1	15.6	15.28	0.19	1.27
		60°	− 0.27	14.6	15.5	14.96	0.32	2.14
		30°	− 1.33	14.1	15.5	14.80	0.52	3.53
Nippon Piston Ring	20	90°	1.20	19.8	20.8	20.24	0.34	1.70
		60°	− 4.90	18.6	19.6	19.02	0.33	1.71
		30°	7.10	19.4	23.4	21.42	1.31	6.12
THOMMEN	15	90°	10.40	15.6	17.4	16.56	0.63	3.83
		60°	4.53	15.3	16.0	15.68	0.28	1.78
		30°	2.13	14.5	16.0	15.32	0.58	3.77
SHOFU	10	90°	45.20	14.1	15.1	14.52	0.34	2.32
		60°	11.80	10.5	11.3	11.18	0.67	5.95
		30°	− 19.20	7.5	8.8	8.08	0.44	5.45

## Discussion

### The effect of the part of the beam aligned to the scale

**Fig. 7:**
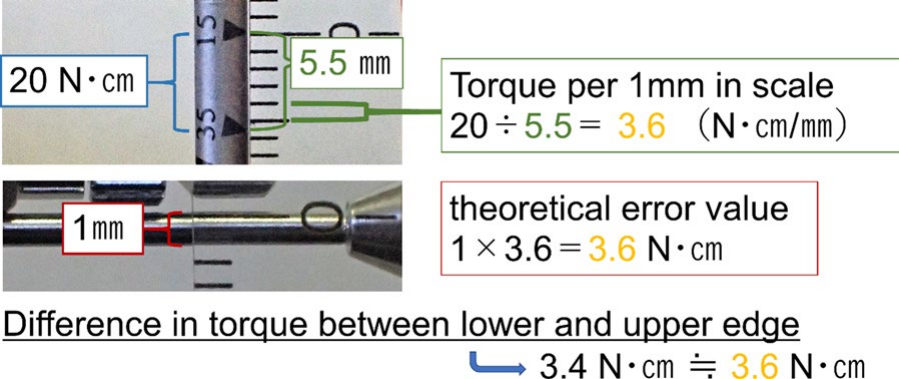
Consideration of the effect of the part of the beam aligned with the scale

Correct torque application on implant components has been a problem since the early days of modern implant dentistry [14, 16, 27–29]. Moreover, studies have shown that in an industrial torque wrench, the magnitude of the volitional torque is strongly influenced by the type of tool used and the posture assumed accordingly [26]. Similarly, in oral implant torque wrenches, the torque is likely to be influenced by the structure of the wrench and the operator’s posture. The structure is thought to be influenced by the beam and the position of the scale pointer. In this study, the torque applied was significantly more accurate when the scale was aligned to the center of the beam. Although no significant difference was observed, reproducibility was highest when the scale was aligned with the center of the beam for the wrenches of many manufacturers. It was hence suggested that the scale should be set at the center of the beam when handling torque wrenches. Additionally, the torque wrenches were greatly affected by the part of the beam that was adjusted to the scale. Thus, it was concluded that the part of the beam adjusted to the scale and the structure of the torque wrench may be related to each other. The structures of the torque wrenches were further analyzed to clarify the relationship between the structure of the torque wrenches and part of the beam to be aligned with the scales. The beam was a cantilever, so bending was not linear and was governed by an equation [30]; thus, the linear scale used for markings was not accurate but could be approximated. In one of the torque wrenches used in this study, the distance between the 35 N cm marking and the 15 N cm marking was 5.5 mm, resulting in a torque value of 3.6 N cm per millimeter. The theoretical value of the error at the trailing and leading edges of the beam could be calculated since the width of the beam was 1 mm. It was suggested that the width of the beam, which is the structure of the torque wrench, was influenced by the part of the beam that is adjusted to the scale (Fig. 7). The correlation coefficients suggested that the torque value per millimeter of the scale and the width of the scale line significantly affected the accuracy, and that the width of the line in turn significantly affected the repeatability. Based on the results of the present study, it can be concluded that it is desirable to adjust the scale to the center of the beam and consider both the influence of the torque value per millimeter of the scale and the width of the beam for each wrench.

### The angle at which the examiner read the torque value

**Fig. 8:**
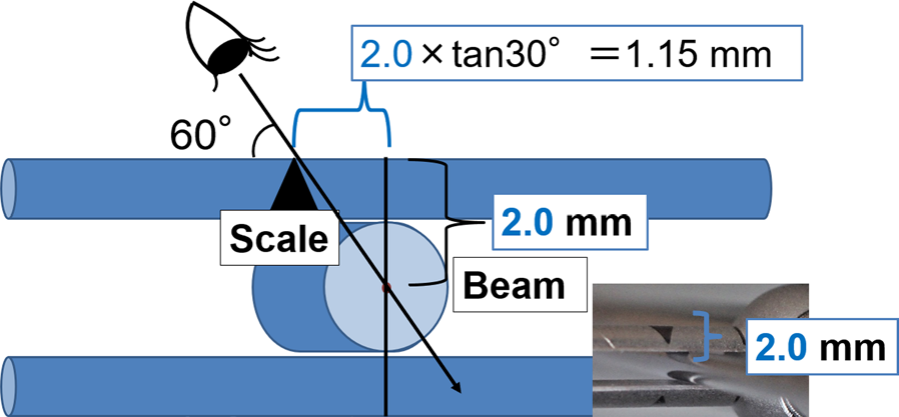
Consideration of the effect of the angle at which the examiner reads the torque value

The torque value was significantly more accurate when the examiner read it from a 90° angle than from a 60° angle, and was significantly more reproducible when read from 90° than from 30°. The accuracy and repeatability were both highest at a reading angle of 90° for the wrenches of most manufacturers. Thus, it was suggested that the torque wrench should be read and used at 90°. Additionally, torque values were greatly affected by the angle at which the examiner read them. Thus, it was suggested that the effect of the angle at which the examiner reads the torque value might be related to the structure of the torque wrench. The structures of the torque wrenches were further analyzed to clarify their relationship with the angle at which the examiner read the torque value. In one of the torque wrenches used in this study, the distance between the scale and the center of the beam was 2 mm, and the distance between the point where the scale was read from 60° and the actual point perpendicular to the center of the beam was 1.15 mm. The torque value per mm was 3.6 N cm, as mentioned previously. The theoretical value of the error was calculated by multiplying the torque value per mm and the distance between the point at which the scale was read from 60° and the vertical point at the center of the beam; thus, it was suggested that the distance between the center of the scale and the center of the beam was a factor affecting the angle (Fig. 8). The correlation coefficients suggested that the distance between the scale and the center of the beam significantly affected the accuracy, and that the torque value per millimeter of the scale and the width of the scale line significantly affected the repeatability. Therefore, the present study indicates that it is desirable to adjust the scale to the center of the beam and consider the influence of the torque wrench after considering the effects of the distance between the scale and the center of the beam, the width of the scale line, and the torque value per millimeter of the torque wrench scale for each manufacturer.

### Strengths and weaknesses in relation to other studies

Neugebauer et al. determined and compared the accuracy of manual wrenches, which were available in different designs with a large range of preset torques. Three different designs were available, with a spring-in-coil or toggle design as an active mechanism or a beam as a passive mechanism to select the preset torque. Beam wrenches are associated with a lower risk of extreme values because of their passive mechanism of achieving the selected preset torque, which minimizes the risk of harming screw connections [31]. In a previous study, we found that beam-type torque wrenches were more accurate than other types of torque wrenches. We studied this aspect further in this study and found that the accuracy and repeatability of the beam-type torque wrench depended greatly on its structure. Regarding the details of the structure, we found that the torque value per millimeter of the scale, the width of the scale line, and the distance between the scale and the center of the beam affected the accuracy, while the torque value per millimeter of the scale and the width of the scale line greatly affected the repeatability.

### Limitations of this study and future perspectives

A limitation of this study is that the torque was measured by a single examiner; therefore, inter-examination measurement error was not considered accordingly. Additionally, since a single torque wrench from each manufacturer was used for the measurements, individual differences were not anticipated in the study. This factor should be considered in future studies.

In the clinical environment, the accuracy and repeatability of torque wrenches change owing to metal fatigue, aging deterioration due to sterilization and cleaning, and wet conditions in the oral cavity. Thus, the effect of aging on the accuracy and repeatability of torque wrenches and the prognostic effect of errors in tightening torque values must be clarified in future studies.

## Conclusion

We suggest that the torque wrench should be read from 90° to the center of the beam aligned to the scale. As observed, accuracy and repeatability differed among the wrenches from different manufacturers. This was related to the torque value per millimeter of the scale, width of the scale line, width of the beam, and distance between the scale and the center of the beam. Thus, a torque wrench must be selected based on the manufacturer’s understanding of the structural differences.

## Data Availability

The datasets used and/or analyzed during the current study are available from the corresponding author upon reasonable request.
